# Intra-arterial Thrombolysis for Postoperative Digital Ischemia: A Case Report

**Published:** 2014-07-11

**Authors:** Johnny I. Efanov, Andrei Odobescu, Marie-France Giroux, Patrick G. Harris, Michel A. Danino

**Affiliations:** ^a^Plastic and Reconstructive Surgery, University of Montreal Hospital Center, Montreal, Quebec, Canada; ^b^Division of Interventional Radiology, University of Montreal Hospital Center, Montreal, Quebec, Canada

**Keywords:** digital ischemia, iatrogenic, intimal injury, subacute, thrombolysis

## Abstract

**Objective:** Surgical repair of digital flexion deformities can result in vascular injuries threatening the viability of the affected digit. While uncommon, these injuries are reported to have a rate as high as 0.8% following palmo-digital fasciectomy for Dupuytren's disease. Late presentation of such vascular events pose a challenge, since taking the patient to the operating room does not guarantee success. **Methods:** We report a case of subacute digital ischemia that presented 10 days following correction of a boutonniere deformity treated with intra-arterial thrombolysis. There were no particular intraoperative complications. The thrombolytic regimen consisted of Alteplase (Roche, Mississauga, Canada) 2 mg bolus and 1 mg per hour (total 30 mg received over 28 hours) and intravenous heparin with a subtherapeutic target partial thromboplastin time of 40 to 50 seconds. **Results:** Thirty hours after the initiation of thrombolysis, an angiography confirmed complete reperfusion of the digital arteries at the distal interphalangeal joint that correlated with the clinical appearance of the digit. Thrombolysis was interrupted and therapeutic intravenous heparin was maintained. Bridging to warfarin was started 6 days postthrombolysis with a target international normalized ratio of 2 to 3. Unfortunately, she was weaned from the heparin while her international normalized ratio was not yet in the therapeutic range and the vessels rethrombosed. This was confirmed by angiography, and intra-arterial thrombolysis was performed with successful revascularization. The patient was restarted on therapeutic dose of heparin and carefully bridged to Coumadin. **Conclusions:** For traction injuries, thrombolytic therapy can be a viable option although we should keep in mind that it could provoke severe adverse events.

Surgical repair of digital flexion deformities can result in vascular injuries threatening the viability of the affected digit. While uncommon, these injuries are reported to have a rate as high as 0.8% following palmo-digital fasciectomy for Dupuytren's disease,^1^ thus exposing hand surgeons to this problem occasionally. Typically, acute ischemia of the digits after repair of severe flexion contractures are detected intraoperatively, leading to immediate correction of the vascular complications, tailoring the treatment to the mechanism of low perfusion, be it vasospasm, thrombosis, vessel laceration, or stretch. Late presentation of such vascular events pose a challenge, since taking the patient to the operating room does not guarantee success. We report a case of subacute digital ischemia that presented 10 days following correction of a boutonniere deformity treated with intra-arterial thrombolysis.

## CASE REPORT

**Figure 1:**
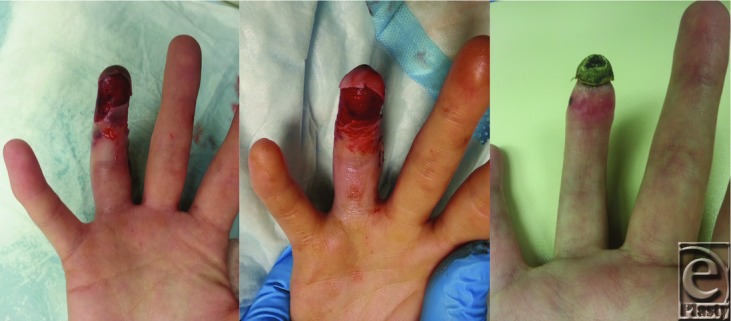
(*Left*) Ischemic finger on presentation showing epidermolysis and a dusky pulp with no capillary return. (*Middle*) Day 1 after thrombolysis. Finger reperfused, with restoration of pink color and a capillary refill at the pulp level. (*Right*) Two months postpresentation. The patient had undergone dry necrosis of the pulp at the level of the third phalanx.

A 25-year-old, right-hand-dominant female patient underwent release of flexion contracture for chronic boutonniere deformity to the ring finger of her dominant hand at another center. According to report of the operating room, the repair was done through a midaxial incision and the digit k-wired in extension. There were no particular vascular trauma or intraoperative complications documented in the report. The patient suffered from a temporary episode of digital ischemia on postoperative day 1, which improved upon K-wire removal. The rapid improvement in perfusion in the subsequent days leads us to believe that this episode was not related to a thrombotic event. However, the patient presented to our institution on postoperative day 10 when she suffered another episode of ischemia. At this time, the examination of the ring finger on the right hand revealed no capillary refill and no bleeding upon puncture (Fig 1, *left*). Patient denied any additional trauma, previous symptoms of thoracic outlet syndrome and had no personal or family history of hypercoagulable disease.

**Figure 2:**
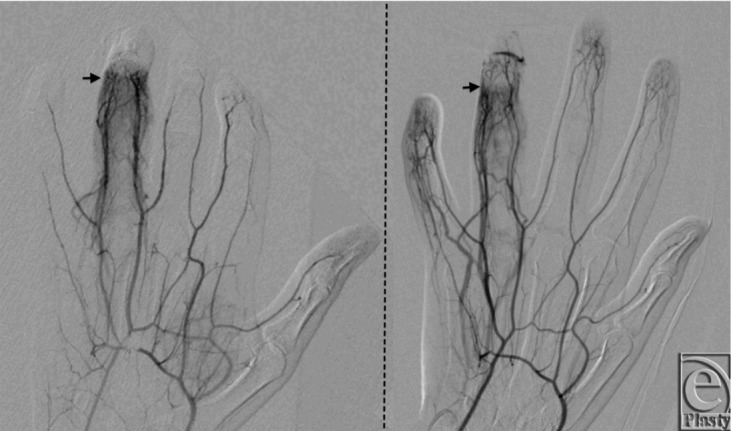
Hand arteriography of the patient's right ring finger showing bilateral occlusion (*arrow*) of digital arteries at the level of the second phalanx (*left*) with acute angles suggesting a thrombotic cause of ischemia. Resolution of previously seen thrombus and distal reperfusion following 24 hours of thrombolytic therapy (*right*).

Angiography showed absent vascularization in the digital arteries of the affected finger, from the level of the middle of the second phalanx. Vasospasm in this setting was unlikely because episodes of reperfusion would have been visualized during the angiographic study. The inflammatory reaction secondary to ischemia can be visualized through the hyperemic appearance of the vessels distally, as opposed to the adjacent fingers (Fig 2), which also indicates an occlusive disorder rather than vasospasm. The appearance of the digital vessels suggested a thrombotic rather than embolic nature of the occlusion, and after discussion with the patient, intra-arterial thrombolysis was initiated.

The thrombolysis was accomplished through a nontapered 4F Glidecath catheter (Terumo, Tokyo, Japan), inserted at the level of the right femoral artery. Because of the very distal lesions, in a young patient, with very spastic forearm arteries, it was decided to perform the thrombolytic drip with the catheter tip in the brachial artery. The thrombolytic regimen consisted of Alteplase (Roche, Mississauga, Canada) 2 mg bolus and 1 mg per hour (total 30 mg received over 28 hours) and intravenous heparin with a subtherapeutic target partial thromboplastin time of 40 to 50 seconds, as per our in-hospital intra-arterial thrombolysis protocol. (target partial thromboplastin time 60-75 seconds).

Thirty hours after the initiation of thrombolysis, a repeat angiography confirmed almost complete reperfusion of the digital arteries at the distal interphalangeal joint that correlated with the clinical appearance of the digit (Fig 1, *middle*). Thrombolysis was interrupted and therapeutic intravenous heparin was maintained. A hematology consultation was asked to rule out any hypercoagulation disorder.

Bridging to warfarin was started 6 days postthrombolysis with a target international normalized ratio of 2 to 3. Unfortunately, she was weaned from the heparin while her international normalized ratio was not yet in the therapeutic range and the vessels rethrombosed. This was confirmed by angiography, and intra-arterial thrombolysis was performed with successful revascularization. The patient was restarted on therapeutic dose of heparin and carefully bridged to Coumadin.

Upon discharge, the distal pulp of the fingertip underwent necrosis because of the prolonged ischemic time. The thrombolysis nonetheless salvaged the portion distal to the middle of the second phalanx that was ischemic on presentation to our center. The distal pulp was allowed to delineate and it spontaneously amputated itself after hospital discharge.

## DISCUSSION

Dias and Braybrooke^2^ found that vascular disturbances (12%) ranked third for most common postoperative complications for Dupuytren's correction, after numbness (36%) and wound infection (19%). Furthermore, vascular complications include several distinct etiologies, and there are currently no large studies addressing causative mechanisms. Previous authors described case reports for emergency microsurgical revascularization after inadvertent transection of both digital arteries,^3^ ischemia caused by vasospasm^4^ and extensive surgical manipulation and atherosclerotic disease leading to arterial thrombosis in the operated fingers.^5^ Evans et al. reported 3 cases of ischemia of the digits, including a case of boutonniere deformity, which were caused by vasospasm.^5^

Two types of occlusive vascular events exist: embolic and thrombotic. In the case of hand surgery, embolic events are rare and in the case of our patient this etiology did not correlate with the clinical or angiographic findings. An embolus creates a more rounded endoluminal defect or a vessel interruption with a cupule as opposed to a thrombus that appears more commonly as a progressive occlusion.^6^ Another unlikely cause is laceration to the digital arteries, but such injury was not documented in the operative report nor exemplified at angiography and thrombolysis.

Thrombosis caused by traction injury of the vessel is the most likely cause of ischemia in our case. Axial pull on the vessels during the correction can result in endothelial damage. The degree of injury is an important determinant because damage to the intima and media as well as a longer surface of injury likely increases the risk of thrombus formation.^7^ For traction injuries, urgent microsurgical revascularization and grafting is a difficult decision to undertake, as both the longitudinal extent and the severity of the injury are unknown. Therefore, as reported in our patient, thrombolytic therapy can be a viable option in this case.

Antithrombotic prophylaxis is critical in the period of reendothelialization of the injured line, lasting a minimum of 7 days in arteries.^8^ This patient's second thrombus after inadequate weaning from Heparin reiterates the importance of maintaining an antithrombotic environment for the entire length of endothelial repair. This reendothelialization period might in fact be more significant in traction injuries in comparison to local trauma or laceration.

The surgeon should also keep in mind that thrombolysis is not without risk and could provoke severe adverse events, such as bleeding (especially worrisome with intracranial hemorrhage),^9^ allergic reactions, hypotension, or menorrhagia. The extensive radiographic exposure, with our patient requiring 3 separate loads of intravenous contrast for the angiographic studies, should also be considered in the decision-making process. While considering the adverse effects and the cost of this management option, a careful assessment of the risks and benefits of angiography and thrombolysis should be discussed with the patient.

We report our experience with a case of subacute ischemia of a digit after repair of a boutonniere deformity that was treated with thrombolysis. The patient's second episode of thrombus formation after stopping heparin prematurely demonstrates the need for thromboprophylaxis for the entire length of endothelial repair, with adequate bridging to Coumadin.
